# University of Louisville—Medical Department

**Published:** 1857-08

**Authors:** 


### UNIVERSITY OF LOUISVILLE--MEDICAL DEPARTMENT.

As will be perceived, the medical department of the University of Louisville will be in operation as usual on the 1st of November next.—The College edifice will be completed and furnished in superior style by the 1st of October.

